# Identification and Enzymatic Activity Evaluation of a Novel *CYP2C9* Allelic Variant Discovered in a Patient

**DOI:** 10.3389/fphar.2021.619339

**Published:** 2021-02-11

**Authors:** Xiao-Yang Zhou, Xiang-Ran Lu, Ying-Hui Li, Ya-Qing Ma, Shi-Wen Zhao, Fang Wang, Ren-Ai Xu, Guo-Xin Hu, Jian-Ping Cai

**Affiliations:** ^1^ The Key Laboratory of Geriatrics, Beijing Institute of Geriatrics, Beijing Hospital, National Center of Gerontology, National Health Commission, Beijing, China; ^2^ Institute of Geriatric Medicine, Chinese Academy of Medical Sciences, Beijing, China; ^3^ School of Pharmacy, Wenzhou Medical University, Wenzhou, China; ^4^ Department of Pharmacy, Zhejiang Yueqing People’s Hospital, Yueqing, China; ^5^ Department of Anesthesiology, Beijing Hospital, National Center of Gerontology; Institute of Geriatric Medicine, Chinese Academy of Medical Sciences, Beijing, China; ^6^ Department of Cardiology, Beijing Hospital, National Center of Gerontology; Institute of Geriatric Medicine, Chinese Academy of Medical Sciences, Beijing, China; ^7^ Department of Pharmacy, The First Affiliated Hospital of Wenzhou Medical University, Wenzhou, China

**Keywords:** cytochrome P450 2C9, allelic variant, drug metabolism, enzymatic activity evaluation *in vitro*, tolbutamide, losartan, warfarin

## Abstract

Warfarin is a widely prescribed anticoagulant but the doses required to attain the optimum therapeutic effect exhibit dramatic inter-individual variability. Pharmacogenomics-guided warfarin dosing has been recommended to improve safety and effectiveness. We analyzed the cytochrome P450 2C9 (*CYP2C9*) and vitamin K epoxide reductase complex subunit 1 (*VKORC1*) genes among 120 patients taking warfarin. A new coding variant was identified by sequencing *CYP2C9*. The novel A > G mutation at nucleotide position 14,277 led to an amino acid substitution of isoleucine with valine at position 213 (I213V). The functional consequence of the variant was subsequently evaluated *in vitro*. cDNA of the novel variant was constructed by site-directed mutagenesis and the recombinant protein was expressed *in vitro* using a baculovirus–insect cell expression system. The recombinant protein expression was quantified at apoprotein and holoprotein levels. Its enzymatic activities toward tolbutamide, warfarin and losartan were then assessed. It exhibited changed *apparent Km* values and increases of 148%, 84% and 67% in the intrinsic clearance of tolbutamide, warfarin and losartan, respectively, compared to wild-type CYP2C9*1, indicating dramatically enhanced *in vitro* enzymatic activity. Our study suggests that the amino acid at position 213 in wild-type CYP2C9*1 may be important for the enzymatic activity of CYP2C9 toward tolbutamide, warfarin and losartan. In summary, a patient taking high-dose warfarin (6.0 mg/day) in order to achieve the target international normalized ratio was found to have a mutation in the *CYP2C9* gene.

## Introduction

Warfarin is the most frequently prescribed oral coumarin-derived anticoagulant drug for the prevention and treatment of arterial and venous thromboembolism. It has a narrow therapeutic index and high inter-patient variability regarding the dose required to achieve the target international normalized ratio (INR) of 2–3 for most indications (Ageno et al., 2012). This variability is influenced by multiple clinical and genetic factors. In 2007, the United States Food and Drug Administration (FDA) updated the warfarin labeling to include information on its pharmacogenomics for the first time (Gage and Lesko, 2008), allowing the therapeutic warfarin dose to be estimated by using pharmacogenomics data on cytochrome P450 2C9 (*CYP2C9*) and vitamin K epoxide reductase complex subunit 1 (*VKORC1*) single-nucleotide polymorphisms. Subsequently, statements from both the US FDA and the Clinical Pharmacogenetics Implementation Consortium (CPIC) further confirmed the importance of genetic factors when determining optimum warfarin dosing (Klein et al., 2009; Johnson et al., 2017; Drozda et al., 2018). Although about 30 genes involved in warfarin metabolism have been identified, the most important genetic factors are the *CYP2C9* and *VKORC1* genotypes (Dean, 2012), especially in the specific population of Asia (Chen et al., 2014). Consequently, *VKORC1* and *CYP2C9* genotyping has been routinely included in pharmacogenomic algorithms for warfarin dosing, along with the two revisions of the warfarin labeling regarding pharmacogenomics.

Warfarin plays a role in anticoagulation by inhibiting the oxidoreductase (OR) encoded by *VKORC1*. It thereby interferes with the vitamin K-dependent carboxylation of glutamic acid residues in coagulation factors II, VII, IX, and X. In clinical practice, warfarin is used as a racemic mixture of both R and S-enantiomers (Miura et al., 2011). The S-enantiomer exhibits 2–5 times more anticoagulant potency than the R-enantiomer in humans and is primarily metabolized and eliminated to produce inactive hydroxylated metabolites by CYP2C9 (Gage and Lesko, 2008). CYP2C9, a polymorphic hepatic enzyme, can metabolite clinical drugs to inactive metabolites (e.g., tolbutamide) or active metabolites (e.g., losartan). CYP2C9 variants can result in absent, decreased, or increased enzyme activity, as can VKORC1 variants. Therefore, these variants are relevant to the clinical efficacy and adverse effects of drugs. According to a randomized clinical trial of 1,650 patients, pharmacogenomics-guided warfarin dosing reduced the risks of major bleeding, venous thromboembolism, and death, compared to clinically guided dosing (Gage et al., 2017).

In this retrospective study, we analyzed the *VKORC1* and *CYP2C9* genotypes of 120 patients taking warfarin. A novel A > G mutation at nucleotide position 14,277 was identified in exon four of *CYP2C9* in a single patient, resulting in an amino acid substitution from an isoleucine to a valine at position 213 of the CYP2C9 protein (I213V). *In vitro* enzymatic activity assays showed that this mutation significantly increased the intrinsic clearance of the CYP2C9 probe drugs tolbutamide, warfarin and losartan.

## Materials and Methods

### Chemicals and Materials

TaKaRa MiniBEST Whole Blood Genomic DNA Extraction Kit, PrimeSTAR HS DNA polymerase, restriction enzymes, and T4 DNA ligase were purchased from Takara Bio Inc. (Kusatsu, Japan). *Spodoptera frugiperda* (S*f*) 21 cells, fetal bovine serum, Sf-900™ III SFM insect culture medium, Cellfectin II Reagent, and the Bac-to-Bac™ Baculovirus Expression System were purchased from Invitrogen (Carlsbad, CA, United States). Tolbutamide, 4-hydroxy tolbutamide, losartan, losartan carboxylic acid, warfarin, 7-hydroxy warfarin, diazepam and midazolam (internal standard [IS]) were purchased from Toronto Research Chemicals (Toronto, Canada). Rabbit polyclonal anti-CYP2C9 antibody and mouse monoclonal anti-OR antibody were obtained from Abcam (Cambridge, United Kingdom) and Santa Cruz Biotechnology (Santa Cruz, CA, United States), respectively. A SuperSignal West Pico Trial Kit was obtained from Thermo Scientific (Rockford, IL, United States). A regeneration system for the reduced form of nicotinamide adenine dinucleotide phosphate (NADPH) was obtained from Promega (Madison, WI, United States). High-performance liquid chromatography-grade organic solvents and liquid chromatography–mass spectrometry-grade acetonitrile were purchased from Merck (Darmstadt, Germany). All other chemicals and reagents used were of the highest purity available.

### DNA Extraction and Genotyping

Genomic DNA samples from peripheral leukocytes were acquired from 120 subjects at the Department of Cardiology of Beijing Hospital (2016BJYYEC-049-02). All subjects provided written informed consent. The study was approved by the ethics committee of Beijing Hospital. After extraction of genomic DNA from the whole blood cells using the TaKaRa MiniBEST Whole Blood Genomic DNA Extraction Kit (Takara Bio Inc. Kusatsu, Japan) according to the manufacturer’s instructions, the whole exons of *CYP2C9* were amplified and sequenced according to previously described methods (Dai et al., 2014). Briefly, PCRs were carried out in a volume of 50 µl containing 100 ng of genomic DNA, 0.5U LA Taq (Takara Bio Inc, Kusatsu, Japan), 1 × GC Buffer I, 0.4 mM dNTP mixture and 0.2 µM of each primer. The optimization of reaction conditions were as follows: denaturation at 95°C for 1 min, followed by 30 cycles at 95°C for 30 s, 50–60°C for 30 s, and 72°C for 0.5–4 min and a final extension at 72°C for 5 min. PCR products were then identified by agarose gel electrophoresis and purified with the TaKaRa MiniBEST Agarose Gel DNA Extraction Kit (Takara Bio Inc. Kusatsu, Japan). The products were sequenced using the ABI Prism BigDye Terminator Cycler Sequencing Kit (Applied Biosystems, Foster City, CA, United States). Eventually, the PCR product possessing the mutation was repeatedly sequenced in both directions to confirm. Additionally, the primers used to assess the c.−1639G > A status regarding the *VKORC1* promoter were as follows: 5′-CAG​AAG​GGT​AGG​TGC​AAC​AGT​AA-3′ and 5′-CAC​TGC​AAC​TTG​TTT​CTC​TTT​CC-3’ (Yuan et al., 2005). All acquired *CYP2C9* sequences were aligned with the reference sequence, NG_008385.2 (LRG_1,195) using Lasergene software (version 8.0, DNASTAR, Madison, WI, United States).

### Mutagenesis and Recombinant Expression Vector Construction

CYP2C9 and OR proteins were simultaneously expressed in *Sf*21 insect cells using the Bac-to-Bac™ Baculovirus Expression System that included the pFastBac™ Dual Expression Vector. The dual expression vector pFastBac-OR-CYP2C9*1 was obtained from our previous study (Dai et al., 2013). To construct each *CYP2C9* variant open reading frame (ORF) used in this study, overlap extension polymerase chain reaction amplification was employed, with wild-type CYP2C9*1 as the template. CYP2C9*3 (I359L) has been reported to show reduced enzymatic activities toward substrates such as tolbutamide, losartan, and warfarin (Goldstein, 2001; Joy et al., 2009), so it was used as the negative control for the enzymatic activity assays; the primers for site-directed mutagenesis were the same as in our previous study (Dai et al., 2013). Regarding the novel variant with an A > G mutation at nucleotide position 14,277, the site-directed mutagenesis primers were as follows: forward primers, 5′-tga​gca​gcc​cct​ggG​tcc​agg​taa​ggc-3′ and 5′-gcc​tga​att​cat​gga​ttc​tct​tgt​ggt-3’; reverse primers, 5′-gcc​tta​cct​gga​Ccc​agg​ggc​tgc​tca-3′ and 5′-gaa​cgt​cga​ctc​aga​cag​gaa​tga​agc​a-3’. The resultant amplified products were purified, digested with EcoRI and Sal I, and then ligated into pFastBac Dual Expression Vectors expressing OR in order to obtain the final dual expression vectors.

### Expression of Recombinant CYP2C9 Holoenzymes in *Sf*21 Cells

Recombinant baculovirus expressing CYP2C9 and OR proteins were prepared using the Bac-to-Bac™ Baculovirus Expression System. After infecting *Sf*21 cells with the viruses, the cells were incubated in Sf-900 II SFM culture medium with 4 ng/µl hemin at 27°C for 4 days. The cells were then harvested and centrifuged at 1,600 × g for 5 min. The pellets were resuspended in 100 mM KPO_4_ containing 1 mM ethylenediaminetetraacetic acid (EDTA), 1 mM phenylmethanesulfonyl fluoride, and 0.25 M sucrose, and sonicated for 40 s on ice using a Vibra-Cell™ sonicator (Sonics & Materials, Inc. Newtown, CT, United States) at 25% of its full power. The homogenate was centrifuged at 13,000 × g for 20 min at 4°C, and the supernatant was then collected and ultracentrifuged at 100,000 × g for 1 h at 4°C. The pellet was resuspended in 100 mM KPO_4_ (pH 7.4) containing 20% glycerol and then stored at −80°C.

### Determination of Expression Levels of CYP2C9 Apoenzymes and Holoenzymes and OR Protein

The expression of the CYP2C9 apoenzymes and OR protein was detected by western blotting following standard procedures (Dai et al., 2013). As described in a previous study (Omura and Sato, 1964), the expression of the CYP2C9 holoenzymes was evaluated using reduced carbon monoxide difference spectroscopy (RCODS) in an Evolution 201 system (Thermo Scientific, Rockford, IL, United States).

### Enzymatic Activity Assays Using CYP2C9 Probe Drugs

The enzymatic activity of each microsomal recombinant CYP2C9 variant expressed in the *Sf*21 cells was evaluated using the classical CYP2C9 probe drugs tolbutamide, warfarin and losartan. Optimized reaction conditions (including reaction buffer, enzyme quantity, and incubation time) were performed based on previous studies (Dai et al., 2013; Dai et al., 2015; Wang et al., 2014) with some modifications. Briefly, the incubation mixture comprised 100 mM reaction buffer (pH 7.4) (phosphate buffer used in tolbutamide and losartan reactions, or Tris-HCl buffer applied in warfarin reaction), 2 pmol purified cytochrome b5, 1 pmol recombinant CYP2C9 microsomal fraction, and substrate (50–2000 μM tolbutamide or 0.1–25 μM losartan or 1–100 μM warfarin). This incubation mixture was pre-incubated at 37°C for 5 min. Thereafter, 10 µl NADPH (20 mM) was added and incubated at 37°C for 40 min with gentle shaking. Finally, the mixture was precipitated with 200 μL acetonitrile and 30 μL IS (midazolam (200 ng/µl) used while testing 4-hydroxy tolbutamide and losartan carboxylic acid or diazepam (25 ng/ml) applied when detecting 7-hydroxy warfarin) and centrifuged at 10,000 × g for 10 min at 4°C. The supernatant was used for subsequent measurements. Data represent the mean ± SD of three independent experiments.

The levels of 4-hydroxy tolbutamide, losartan carboxylic acid, 7-hydroxy warfarin, diazepam and midazolam were measured by ultra-high-performance liquid chromatography–tandem mass spectrometry (UPLC-MS/MS). The detection of 4-hydroxy tolbutamide, losartan carboxylic acid and midazolam (IS) was performed using an ACQUITY I-Class UPLC system coupled to a XEVO TQD triple quadrupole mass spectrometer (Waters Corp., Milford, MA, United States), and fitted with an Acquity UPLC BEH C18 column (2.1 × 100 mm, 1.7-μm particle size; Waters Corp.). An ACQUITY I-Class UPLC system coupled to XEVO TQS triple quadrupole mass spectrometer, fitted with an Acquity UPLC BEH C18 column (2.1 × 50 mm, 1.7-μm particle size; Waters Corp.), was used to quantify the level of 7-hydroxy warfarin and diazepam (IS). The mobile phases involved water with 0.1% formic acid (A) and acetonitrile (B), with a flow rate of 0.4 ml/min and an injection volume of 5 µl. A gradient elution program was used to separate the 4-hydroxy tolbutamide and midazolam, as follows: 0–1.4 min (60–10% A) and 1.4–2.6 min (10–60% A). The total run time was 4 min. To separate losartan and midazolam, we used an isocratic elution of 25% A, with a flow rate of 0.5 ml/min; the total run time was 2.5 min. We used another gradient elution program for 7-hydroxy warfarin and diazepam: 0–0.5 min (10–10% B), 0.5–1.0 min (10–90% B), 1.0–2.0 min (90–90% B), 2.0–2.1 min (90–10% B), and 2.1–3.0 min (10–10% B); the total run time was 3 min. The target compounds were detected using positive electrospray ionization based on multiple reaction monitoring transitions of a mass/charge ratio (*m/z*) 287 → 74.02 for 4-hydroxy tolbutamide, a *m/z* of 437.2 → 235.2 for losartan carboxylic acid, a *m/z* of 325 → 178.98 for 7-hydroxy warfarin, a *m/z* of 284.91 → 153.9 for diazepam, and a *m/z* of 326.1 → 291.1 for midazolam (IS). The cone voltage and collision energy were set at 30 V and 15 eV for 4-hydroxy tolbutamide, 35 V and 15 eV for losartan carboxylic acid, 20 V and 15 eV for 7-hydroxy warfarin, 10 V and 30 eV for diazepam, and 60 V and 30 eV for midazolam, respectively. The gas temperature was set to 500°C at a flow rate of 1000 L/h when detecting 4-hydroxy tolbutamide or losartan carboxylic acid, but was set to 600°C at a flow rate of 800 L/h when testing 7-hydroxy warfarin and diazepam.

The enzymatic kinetic parameters, including the Michaelis–Menten constant (*apparent K*_*m*_)*,* and maximum reaction velocity (*V*_max_), were estimated using Prism 5 (GraphPad, San Diego, CA, United States) based on non-linear regression analyses. The intrinsic clearance (CL_int_) was calculated as *V*_max_ */apparent K*_*m*_. The enzymatic activity of the wild-type and variant CYP2C9 was compared by one-way analysis of variance with Dunnett’s post-hoc test using SPSS software (version 16.0, SPSS Inc., Chicago, IL, United States), with *p* < 0.05 representing statistical significance.

## Results

### Identification of a New *CYP2C9* Allelic Variant

**FIGURE 1:**
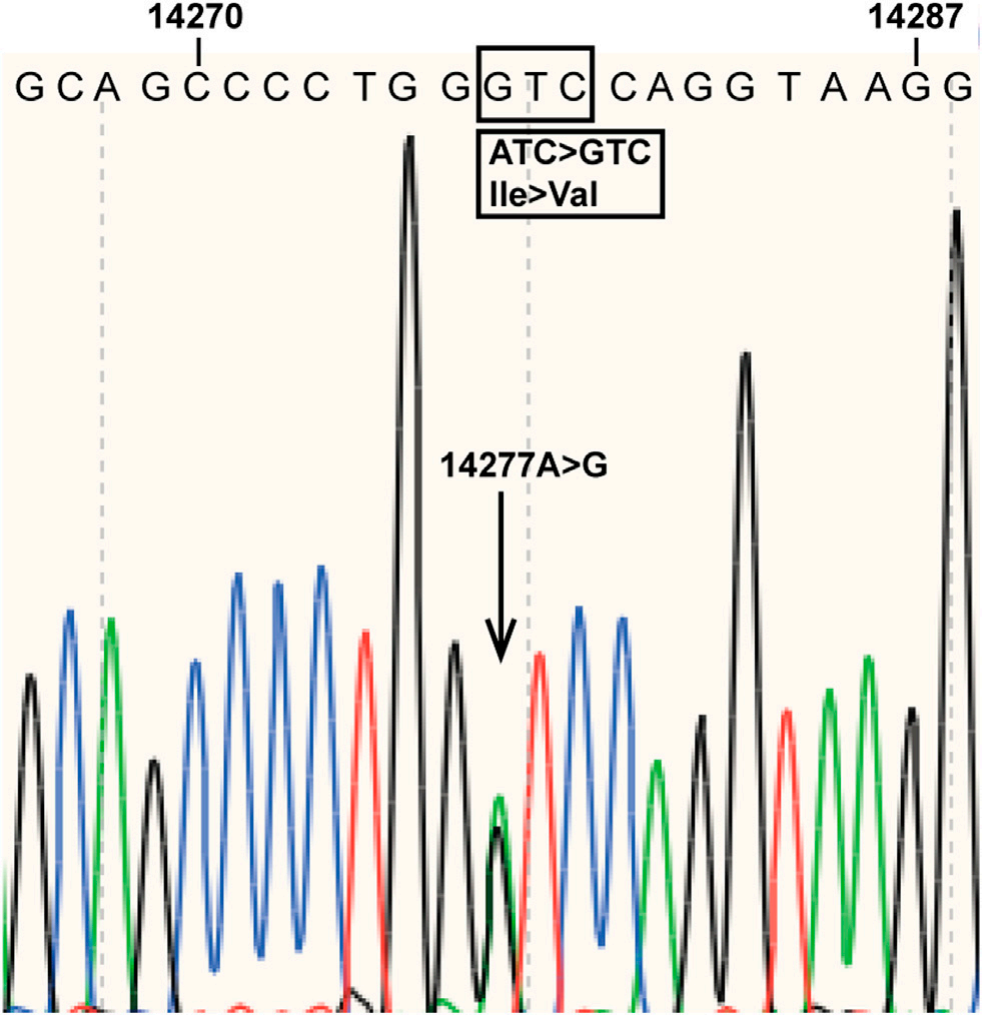
Novel mutation in *CYP2C9* exon 4. The arrow indicates the mutation detected at nucleotide position 14,277 of the reference sequence (NG_008385.2). The boxes show the mutation and the amino acid substitution (I213V) caused by the mutation.

During an investigation of pharmacogenomics-guided warfarin dosing among 120 randomly selected Chinese patients taking warfarin at the Department of Cardiology of Beijing Hospital, the subjects underwent *CYP2C9* and *VKORC1* genotyping. A novel *CYP2C9* mutation, A > G at nucleotide position 14,277, was found in a patient with atrial fibrillation and diabetes. The patient was a 72-year-old Han Chinese female who had been taking high-dose (6.0 mg/day) warfarin to achieve an INR of 2.0–3.0 in order to prevent thrombogenesis. The novel mutation was located in *CYP2C9* exon 4, resulting in an amino acid substitution at position 213 of the protein (I213V) (Figure 1). The data for the study have been deposited in the European Nucleotide Archive (ENA) at EMBL-EBI under accession number PRJEB40955 (https://www.ebi.ac.ul/ena/browser/view/PRJEB40955). The novel *CYP2C9* mutation has also been submitted to the PharmVar Consortium (https://www.pharmvar.org/gene/CYP2C9). Regarding the patient’s *VKORC1* c.-1639G > A status, the A/A genotype was detected.

### Expression of the Recombinant CYP2C9 Variants in *Sf*21 Cells

**FIGURE 2:**
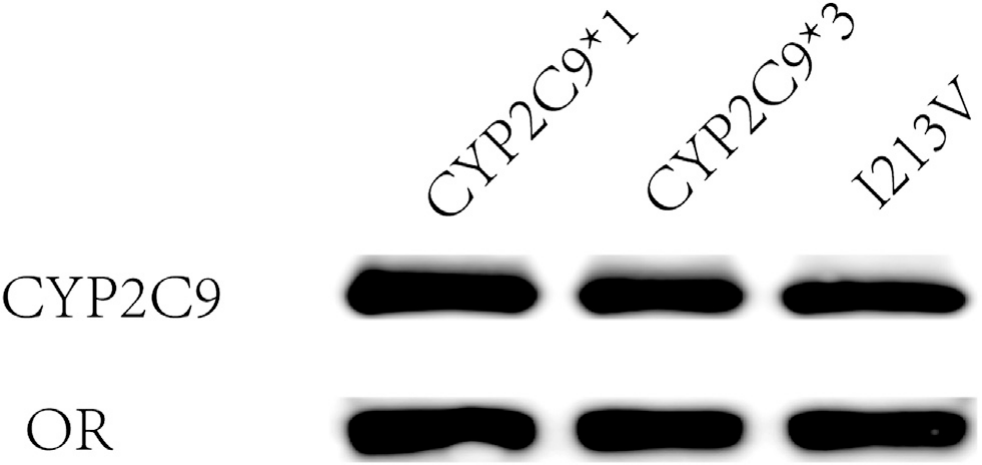
Expression of *CYP2C9* alleles in *Sf*21 insect cells. The externally expressed oxidoreductase (OR) and cytochrome P450 2C9 (CYP2C9) proteins were identified by western blotting with antibodies against OR and CYP2C9, respectively.

**FIGURE 3:**
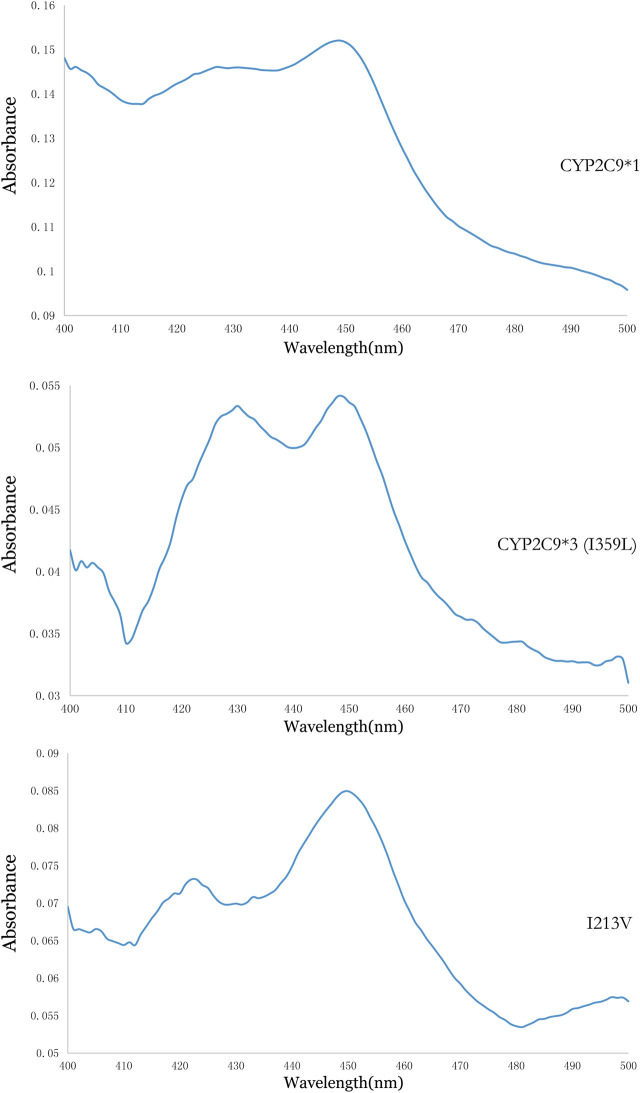
Reduced carbon monoxide difference spectra for the recombinant CYP2C9 in the *Sf*21 insect cell microsomes.

Using methods described in a previous study (Dai et al., 2013), coexpression of CYP2C9 and OR proteins was achieved in *Sf*21 cells. The enzymatic activity of the recombinant novel CYP2C9 variant was then assessed. To conduct the enzymatic assays, recombinant CYP2C9 holoenzymes (the wild-type positive control CYP2C9*1, the negative control CYP2C9*3, and the novel CYP2C9 variant [I213V]) along with the OR protein were successfully coexpressed in the *Sf*21 cells. Western blotting indicated that the expression levels of the three apoenzymes were similar (Figure 2). As shown in Figure 3, the CYP2C9 holoenzymes were quantified using RCODS, which indicated that they possessed CYP2C9 holoenzyme activity. The levels of CYP2C9*1, *3 and I213V were 91, 27, 52 pmol/mg protein, respectively.

### Evaluation of Enzymatic Activities of the Recombinant CYP2C9 Variants

**FIGURE 4:**
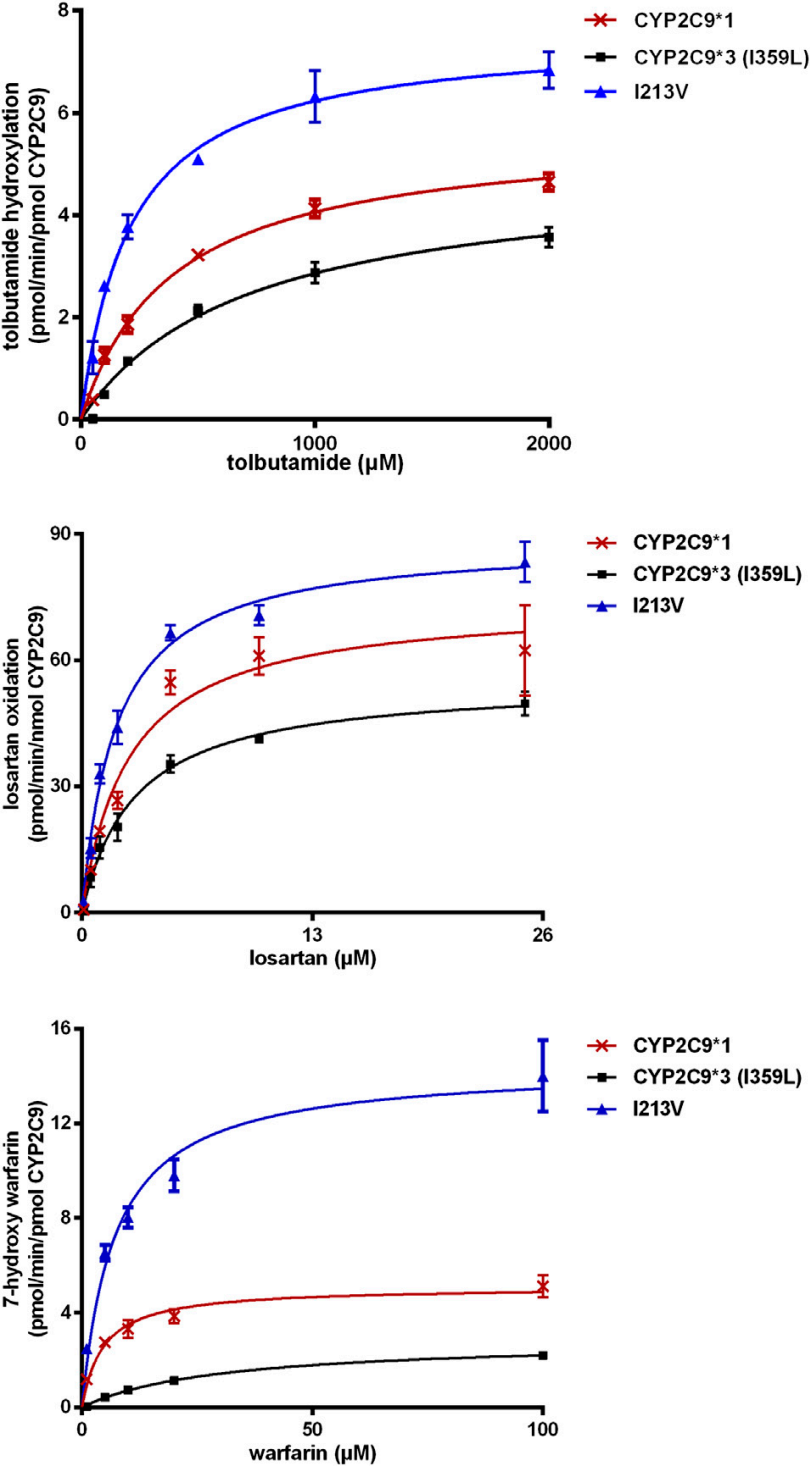
Michaelis–Menten kinetics for tolbutamide hydroxylation, warfarin hydroxylation and losartan oxidation catalyzed by microsomal recombinant CYP2C9. Each point represents the mean ± SD from three experiments.

The enzymatic activities of the recombinant CYP2C9 holoenzymes were evaluated using the three CYP2C9 probe drugs: tolbutamide, warfarin and losartan. The novel CYP2C9 variant (I213V) exhibited significantly changed *apparent K*_*m*_, increased *V*_max_ values, and increased intrinsic clearance of tolbutamide (247.6%), warfarin (183.6%) and losartan (166.5%) compared to wild-type CYP2C9*1. All enzymatic kinetic parameters are shown in Table 1 and Figure 4. Our results suggest that the substitution of isoleucine with valine at position 213 of the protein, resulting from the newly identified A > G mutation at nucleotide position 14,277, enhances the enzymatic activity of the CYP2C9 enzyme toward tolbutamide, warfarin and losartan.

**TABLE 1:** Kinetic parameters for tolbutamide hydroxylation, warfarin hydroxylation and losartan oxidation catalyzed by microsomal recombinant CYP2C9.

CYP2C9 variant	Tolbutamide				Losartan			
	V_max_	Apparent K_m_	Intrinsic clearance (V_max_/apparent K_m_)	Relative clearance	V_max_	Apparent K_m_	Intrinsic clearance (V_max_/apparent K_m_)	Relative clearance
	pmol/min/pmol CYP2C9	μM	μL/min/nmol CYP2C9	%*^a^*	pmol/min/nmol CYP2C9	μM	μL/min/nmol CYP2C9	%*^a^*
CYP2C9*1 (wildtype)	5.6 ± 0.1	390.8 ± 29.2	14.5 ± 0.9	100.0	74.3 ± 6.9	2.7 ± 0.5	27.4 ± 2.6	100.0
CYP2C9*3 (I359L; negative control)	4.9 ± 0.3*	715.5 ± 90.8	6.9 ± 0.4*	47.8	55.3 ± 3.3*	3.1 ± 0.7	18.5 ± 3.4*	66.9
Novel CYP2C9 variant (I213V)	7.5 ± 0.4*	211.4 ± 4.1*	35.7 ± 2.4*	247.6	88.5 ± 2.5*	1.9 ± 0.1	45.7 ± 1.6*	166.5

CYP2C9 variant	Warfarin			
	V_max_	Apparent K_m_	Intrinsic clearance (V_max_/apparent K_m_)	Relative clearance
	pmol/min/pmol CYP2C9	μM	μL/min/pmol CYP2C9	%*^a^*
CYP2C9*1 (wild-type)	5.1 ± 0.5	4.7 ± 1.1	1.1 ± 0.2	100.0
CYP2C9*3 (I359L; negative control)	2.9 ± 0.1*	30.2 ± 0.6*	0.1 ± 0.0*	8.2
Novel CYP2C9 variant (I213V)	14.5 ± 1.7*	7.2 ± 1.4*	2.0 ± 0.2*	183.6

Data represent mean±SD (unless otherwise stated) from three experiments.

^a^Relative to wild-type CYP2C9*1.

*p< 0.05 *vs.* wild-type CYP2C9*1.

## Discussion

We found a novel mutation in exon four of the *CYP2C9* gene in a patient taking warfarin. The A > G transition at nucleotide position 14,277 results in an amino acid mutation at position 213, i.e., the substitution of isoleucine with valine. The patient was heterozygous regarding *CYP2C9*, with wild-type CYP2C9*1 and the novel *CYP2C9* variant (I213V). As far as we know, this variant might be the first gain-of-function allele found in a patient and has not previously been submitted for inclusion in the nomenclature database for CYP450 alleles managed by the PharmVar Consortium. CYP2C9 is one of the most polymorphic enzymes, with 62 allelic variants identified and named so far by the PharmVar Consortium. In humans, it metabolizes approximately 20% of clinical drugs in the liver (Mo et al., 2009), including warfarin, diclofenac, tolbutamide, and losartan, as the main phase I enzyme. Tolbutamide and losartan have been commonly used as probe substrates for CYP2C9 (Lee et al., 2003). Tolbutamide is a better CYP2C9 probe drug than losartan because its formation clearance (involving CYP2C9-mediated production of tolbutamide-derived metabolites) is more strongly associated with genotype than that of losartan (Lee et al., 2003). In this study, tolbutamide and losartan along with warfarin were all used to assess the enzymatic activity of the novel variant.

Our enzymatic assays indicated that the novel mutation increased the intrinsic clearance of tolbutamide, warfarin and losartan by 148%, 84%, and 67% (Table 1), respectively. Compared to wild-type CYP2C9*1, the CYP2C9 variant (I213V) exhibited a decrease in *apparent Km* regarding tolbutamide and losartan of 50% and 30%, respectively, but an increase in *apparent Km* for warfarin, which indicates a higher affinity for both tolbutamide and losartan, but a lower affinity for warfarin. That is, the novel variant with a mutation in the coding region of the gene possesses variable preference and enhanced enzymatic activity toward tolbutamide, warfarin and losartan compared to wild-type CYP2C9*1. A study of the crystal structure of human CYP2C9 demonstrated that residues 212–222 form helices F′ and G′ in the F-G loop, which is one of two loops that are widely considered to be involved in the substrate access channel (Williams et al., 2003). Another study on the structure of CYP2C9 in relation to genetic polymorphisms showed that wild-type CYP2C9*1 was observed to bind of an additional losartan (the third losartan) in the substrate access channel. The sidechain of Q214 in wild-type CYP2C9*1 interacted with the imidazole ring of losartan in the access channel near the hydroxyl moiety, the potential activation site of losartan (Maekawa et al., 2017). Interestingly, the same sidechain in the CYP2C9*30 (A477T) rotates more than 90° from the losartan activation site to hydrogen bond (Maekawa et al., 2017), and the variant showed 99% reduced activity (Maekawa et al., 2009). A hydrophobic substitution of leucine at position 214 (in CYP2C9*28) led to decreased enzymatic activity and thus decreasing the formation of the active metabolite of losartan (Furie, 2013). Furthermore, the L208V variant has been reported to selectively increase the 6-hydroxylation of S-warfarin, indicating that the F-G loop region of CYP2C9 may influence the substrate orientation in the active site (Mosher et al., 2009). Obviously, although these residues in the F-G loop are outside the active site, they are able to affect substrate binding and protein conformation in the F-G region (Maekawa et al., 2017). Consequently, it is reasonable to presume that a substitution of isoleucine with valine at position 213 in the F-G loop influences substrate binding and protein conformation and, thus, enzymatic activity.

Genetic variation in *VKORC1* is also an important factor that should be considered when determining the appropriate warfarin dosing. A common polymorphism in the promoter region of *VKORC1*, c.-1639G > A, is believed to change a transcription factor-binding site, decreasing the protein expression, so patients with at least 1 A allele tend to require lower initial and maintenance doses of warfarin compared to those with a G/G genotype (Yuan et al., 2005; Gage and Lesko, 2008; Dean, 2012). Notably, the A allele frequency is very high (around 90%) in Asians, which may contribute to the lower warfarin dosing required for Asian patients (Yuan et al., 2005; Dean, 2012). In this study, the Chinese patient with the novel *CYP2C9* mutation had the A/A *VKORC1* genotype*.* The patient had been taking 6.0 mg/day warfarin, achieving a good therapeutic effect, after weekly dose adjustment based on the INR. This study represented a continuation of an earlier project in which we enrolled several hundred elder patients taking warfarin to establish a new prediction algorithm for stable warfarin dose for the elderly Han-Chinese population under the guidance of pharmacogenetics (Ren et al., 2020). The mean stabilized warfarin dose for this population that was applied in the project ranged from 2.39 to 4.06 mg/day. We found that the CYP2C9 wild-type *1*1 and VKORC1 (AA) were most common group (275 cases, 76.39%); the mean stabilized dose of warfarin was 3.0 mg/day (Ren et al., 2020). In addition, we used a prediction model (www.warfarindosing.org) to estimate the initial therapeutic dose of warfarin in a hypothetical patient possessing CYP2C9*1/*1 and VKORC1 A/A genotype. The clinical factors of the hypothetical patient required in the model were consistent with those of the patient detected in the study carrying the I213V mutation, including age, sex, ethnicity, weight, height, smoking status, indications, INR, and drug combination. The estimated initial therapeutic dose of warfarin was 3.0 mg/day for the hypothetical patient, according to the algorithm based on clinical factors and the CYP2C9 and VKORC1 genotypes (Gage et al., 2008). The mean stable dosage required for the elderly Chinese patients identified in Ren’s study (Ren et al., 2020) were similar to the estimated initial dose shown above. However, this dose was far lower than the actual dosage of warfarin used for the patient possessing the I213V mutation. Combined with our results, the *in vitro* enzymatic activity of the variant (I213V) toward warfarin led us to speculate that the novel *CYP2C9* mutation (I213V) found in the patient was likely to represent a gain-of-function mutation. The high dosage of warfarin required to achieve the target INR for the patient may be explained by taking into account both clinical factors and the novel CYP2C9 variant (I213V) which dramatically enhanced the *in vitro* enzymatic activity of CYP2C9.

In summary, a novel A > G mutation at nucleotide position 14,277 in *CYP2C9* was identified, giving rise to a substitution of isoleucine with valine at position 213 in the protein. The recombinant variant exhibited dramatically increased intrinsic tolbutamide, warfarin and losartan clearance *in vitro*. The hypothesis that the novel variant affects the warfarin dosage required to achieve an optimum therapeutic effect remains to be validated in future studies.

## Data Availability

The datasets presented in this study can be found in online repositories. The names of the repository/repositories and accession numbers can be found below: https://www.ebi.ac.uk/ena, PRJEB40955.

## Abbreviations

CPIC, Clinical Pharmacogenetics Implementation Consortium; CYP2C9, Cytochrome P450 2C9; FDA, Food and Drug Administration; IS, internal standard; OR, Oxidoreductase; ORF, Open Reading Frame; PharmVar Consortium, Pharmacogene Variation Consortium; RCODS, reduced carbon monoxide difference spectroscopy, VKORC1, Vitamin K epoxide reductase complex subunit 1; UPLC-MS/MS, ultra-high-performance liquid chromatography–tandem mass spectrometry
